# Solid Pro-Nano Lipid Oral Formulations for Cannabidiol (CBD)

**DOI:** 10.3390/pharmaceutics18040436

**Published:** 2026-03-31

**Authors:** Awanish Kumar, Ayala Bar-Hai, Muhammad AbdEl-haq, Michal Gur, Amnon Hoffman, Abraham J. Domb

**Affiliations:** 1 Institute of Drug Research, School of Pharmacy, Faculty of Medicine, The Hebrew University of Jerusalem, Jerusalem 91120, Israelamnonh@ekmd.huji.ac.il (A.H.); 2 The Core Research Facility, Faculty of Medicine, The Hebrew University of Jerusalem, Jerusalem 91120, Israel; 3 Jerusalem College of Technology (JCT), Machon Lev, Jerusalem 91160, Israel

**Keywords:** solid pro-nano lipids (SPNL), liquid PNL (LPNL), cannabidiol (CBD), long-term stability, pharmacokinetics (PK), oral bioavailability

## Abstract

**Background**: Solid pro-nano lipid (SPNL) oral formulations were prepared and tested in rats for enhanced oral bioavailability of cannabidiol (CBD). **Methods**: The solid formulation at room temperature is a uniform solution of CBD in a mixture of solid lipids and surfactants. Upon contact with aqueous media, it disperses into <200 nm particles. Up to 40% *w*/*w* of CBD can be loaded in this formulation into a hard gelatin capsule or mixed with solid additives and compressed into a tablet. Another type of SPNL formulation was prepared from the absorption of a liquid pro-nano lipid formulation onto a solid support, termed LPNL. **Results**: Pharmacokinetic studies on male Wistar rats (0.295–0.335 kg) reveals that a single oral dose of SPNL or LPNL leads to rapid CBD absorption and high C_max_ values. The SPNL and LPNL formulations are stable at room temperature for at least 3 months. Powder forms of the SPNL and LPNL were prepared with Neusilin US2, SYLOID 244 FP, microcrystalline cellulose (Avicel PH 102), and mannitol. Both SPNL and LPNL show lesser stability for CBD with mesoporous silica particles such as Neusilin US2 and SYLOID 244 FP. **Conclusions**: The SPNL formulations do not contain any organic solvent and therefore are safer compared to the SNEDDS systems. These solid lipids-based oral formulations can be applied for the delivery of other lipophilic drugs.

## 1. Introduction

The pharmaceutical recognition of cannabidiol (CBD) is growing. Numerous pharmacological studies have appeared in the literature, announcing new potential uses of CBD due to its anti-inflammatory and neuropharmacological properties, including antiepileptic, sedative, anxiolytic, and antipsychotic activities. The bioavailability of the CBD is higher when the CBD is taken together with a high-fat meal [1]. The high lipophilicity (logP 6.3) of CBD makes it more soluble in lipids and oils. Upon ingestion, it initiates micelle formation from hydrolyzed lipids that aid in the bio-accessibility of CBD [2]. Oral delivery of CBD in the form of Epidiolex^®^ is the only FDA-approved medication. The composition of Epidiolex^®^ includes 10% CBD in a mixture of sesame oil (primarily containing triglycerides) and anhydrous ethanol [3]. However, to obtain better oral bioavailability, a relatively high dose of Epidiolex^®^ is needed, and this is associated with gastrointestinal side effects [4]. Therefore, there is a growing demand for better CBD formulations having faster bioavailability relative to Epidiolex^®^.

Self-nanoemulsifying drug delivery systems (SNEDDS) are formulations that are designed to increase the solubility, stability, and bioavailability of lipophilic drugs. SNEDDS formulations are mixture of oils, surfactants, and co-surfactants that self-emulsify to form nano-emulsions (<200 nm) upon contact with water [5,6]. These nano-emulsions provide a larger interfacial surface area due to their small droplet size, which in turn may have impact on drug absorption in the GI tract [7]. SNEDDS formulations improve the solubility and stability of CBD. Recent SNEDDS formulations have shown improvement in the oral absorption of CBD [8,9,10,11,12,13]. We have reported on pro-nano liposphere formulations (PNL) for CBD, which are modified versions of SNEDDS formulations. The uniqueness that differentiates PNL from the SNEDDS formulations is in its instant formation of semisolid-core nanoparticles in aqueous dispersion [14,15,16]. The bioavailability of CBD in healthy volunteers shows a 4-fold increase of CBD in the blood after oral administration of PNLs [17].

The SNEDDS and PNL formulations are all in liquid form. The stability of such liquid formulations is subject to severe drawbacks. Complex challenges in the handling and storage of SNEDDS formulations often occur, resulting in drug precipitation and phase separation of the components. This instability in the liquid SNEDD formulations is irreparable and often does not yield the required emulsion droplet size [18]. Compatibility issues could also arise while filling the SNEDDS into soft shell capsules. This contributes to higher manufacturing costs [19,20].

The stability of liquid SNEDDS can be increased by absorbing it into solid carriers [21]. This approach converts SNEDDS into a free-flowing powder (solid-SNEDDS) that can be compressed into oral tablets or can be filled easily into capsules. Buya et al. [22] reviewed the literature and listed the most-used solid carriers, such as silicates, silicon dioxide (i.e., Aerosil^®^), sodium carboxymethylcellulose, micronized porous silica (i.e., Neusilin US2, SYLOID 244, etc.), hydroxylpropyl beta cyclodextrin, and magnesium stearate. Lactose, starch, mannitol, calcium carbonate, crosslinked sodium carboxymethyl cellulose, cross-linked polymethyl methacrylate, and crospovidone have also been reported as carriers for SNEDDS [23]. CBD-loaded SNEDDS powder was obtained by absorption onto Fujicalin^®^, (porous particles of dibasic calcium phosphate) [8]. One challenge is to find the correct solid carrier for liquid SNEDDS or PNLs. Not all available solid carriers are suitable and sufficiently compatible to absorb liquid SNEDDS [18]. Beg et al. [24] note the “squeezing out” of the absorbed liquid from solid-SNEDDS during compaction and compression, leading to failure of tablets in a hardness test. Also, reports show that not all drugs are released from the solid-SNEDDS in an aqueous medium, which narrows the selection of solid carriers for CBD [8].

The problems associated with the preparation of the solid-SNEDDS systems can be reduced with our newly developed solid pro-nano lipid oral formulations (SPNL). We propose the development of PNL systems that physically appear as solid wax at room temperature. The SPNL are mixtures of a solid lipid, oils, surfactants, and co-surfactants that self-emulsify in an aqueous medium to form the desired nanoparticles of <200 nm under modest agitation like that seen in the gastrointestinal tract. CBD solubility in SPNL is up to 40% *w*/*w*, which is considerably higher than the SNEDDS or our previously reported PNL formulations. The stability of CBD in SPNL is very commendable at room temperature. For comparison with SPNL, we also present the modified version of our previously reported liquid PNLs [15]. The new liquid PNL formulation (abbreviated as LPNL) is a liquid capable of holding 25% *w*/*v* CBD. Neither the SPNL nor the LPNL contain any organic solvents such as ethanol and ethyl lactate. We further analyzed the oral bioavailability of the SPNL and LPNL from the pharmacokinetics (PK) data in the rats.

## 2. Materials and Methods

### 2.1. Materials

Cannapure^®^ PH (Cannabidiol) (LOT number 10300024) was obtained from Symrise AG, 37603 Holzminden, Germany. Curcumin (#CAS 458-37-7) and 7-Nitroindazole (7-NI) (#CAS 2942-42-9) were purchased from ThermoFisher Scientific, Waltham, MA 02451, USA. Ibuprofen (#CAS 15687-27-1) was obtained from Ethyl Corporation, OrangeBurg, SC, USA. Sesame oil (#CAS 8008-74-0) was obtained from Henry Lamotte Oils GmbH, 28197 Bremen Germany. Kolliphor^®^RH 40 (a copolymer of ethylene oxide and hydrogenated castor oil at a 40:1 mole ratio) ((#CAS61788-85-0) and Kolliphor^®^ HS 15 (Solutol HS 15) (Macrogol-15-hydroxystearate) (#CAS 70142-34-6) were obtained from BASF CORPORATION, NJ 07932, USA. MONTANOX™ 80 (Tween 80) (9005-65-6) and MONTANE™ 80 (Span 80) (#CAS 1338-43-8) were obtained from SEPPIC, La Garenne-Colombes, Île-de-France 92250. Propylene glycol (#CAS 57-55-6) and Tween 20 (#CAS 9005-64-5) were obtained from Sigma Aldrich, USA. IMWITOR^®^ 900 K (Glycerol Monostearate 40–55%, Type II) (#CAS 85251770) was received as a generously provided sample from IOI Oleo GmbH, Hamburg, Germany. Labrafil^®^ M 1944 CS (Oleoyl macrogol-6 glycerides) and Geleol™ Mono and Diglycerides NF (Glyceryl Monostearate) (#CAS 85251770) were received as generously provided samples from Gattefosse, 69800 Saint-Priest, France. Neusilin^®^ US2 and Fujicalin^®^ (dibasic calcium phosphate porous particles) were received as generously provided samples from Fuji Chemical Industries Co., Ltd., Tokyo 105-0011, Japan. Avicel PH 102 (microcrystalline cellulose) was received as a generously provided sample from BSC Distributors Ltd., 4673327, Herzelia, Israel. SYLOID^®^ 244 FP silica was received as a generously provided sample from GRACE, Columbia, MD 21044, USA. Mannitolium (Mannitol-silicon dioxide (99.5% mannitol and 0.5% silicon dioxide)) filler were obtained from Fagron GmbH & Co., 21509 Glinde, Germany. Mannitol powder was provided by Fertin Pharma, 7100 Vejle, Denmark.

### 2.2. Methods

#### 2.2.1. Preparation of CBD-Loaded PNL Formulations

Blank formulations were prepared using the composition as shown in Table 1. SPNL and LPNL were obtained by heating the mixtures to 85 °C and 37 °C, respectively, for about 30 min until clear solutions were obtained. The compositions were then allowed to cool and stored in closed vials at room temperature until further use. To prepare CBD-loaded SPNL and LPNL formulations, known weights of the CBD were added to the PNLs at 75 °C and 37 °C, respectively, and mixed for about 30 min until clear solutions were obtained.

#### 2.2.2. High-Performance Liquid Chromatography (HPLC) for CBD Quantification

The HPLC system consists of a MERCK HITACHI HPLC system with an INTERFACE D7000, UV DETECTOR L7400, COLUMN OVEN L7360, AUTOSAMPLER L7200 and DEGASSER ERC-3415a. A Luna column (C-18(2), 5 μm, 150 × 4.6 mm, 100 Å Phenomenex, P/No. 00F-4252-E0) was used to quantify the CBD. Specifically, 80% acetonitrile: 20% 5 mM NaH_2_PO_4_ aq. solution (pH 3.0) was the mobile phase, demonstrating a flow rate of 1 mL/min. The sample injection was 20 µL, and the detection wavelength for CBD was 211 nm. The column temperature was set at 35 °C. The CBD retention time (RT) was observed to be 4.8 ± 0.2 min.

Approximately 10 mg of the PNL was placed in a 20 mL glass vial containing 20 mL of methanol and secured by a cap with parafilm. The mixture was sonicated for 40 min in a water bath at room temperature. Only for the SPNL, after sonication, the methanol mixture was kept at 40 °C in water bath for 30 min. The mixture then was vortexed for 2 h at room temperature to ensure the complete extraction of the drug. The methanol mixture with the PNL was filtered with 0.22 µm nylon syringe filters and analyzed using HPLC as stated above.

#### 2.2.3. Dispersion Characterization

Dynamic light scattering (DLS) determined the size of the CBD–PNL dispersion; this was determined with the use of a Zetasizer (Nano ZS, Malvern Instruments, Worcestershire, WR14 1XZ, UK) equipped with inbuilt software. Within the DLS settings, the refractive index 1.47 (absorption: 0.01) was set for the reference material. DDW was chosen as a dispersant (refractive index 1.33, mPa.s 0.8872 at 37 °C). In a typical experiment, 200 mg of CBD-loaded PNL was transferred into a 20 mL glass vial. Then, 5 mL of pre-heated phosphate buffer (PBS, pH 7.2, 37 °C) was added and mixed using a vortex mixer to form a uniform nano-dispersion. Lastly, 20 µL of the CBD–PNL dispersion was further diluted with 1 mL DDW at 37 °C in a DLS cuvette (DTS1070) and checked for size and surface charge (zeta potential) measurements. All measurements were conducted in triplicate at 37 °C for each batch of SPNL or LPNL.

#### 2.2.4. Storage Stability Studies

The stability study was conducted by placing the SPNL or LPNL formulations in tightly sealed bottles for 90 days in the dark at room temperature. Samples were withdrawn at various intervals and analyzed for loss of CBD content using HPLC.

#### 2.2.5. Transmission Electron Microscopy (TEM)

The aqueous dispersions of the SPNL or LPNL formulations were observed using transmission electron microscopy (JEOL TEM JEM-1400, Tokyo 196-8558, JAPAN). For this, 10 µL from the nano-dispersion (1 mg/mL) was deposited on a copper grid, forming a thin liquid film. The film was then negatively stained with 2% (*w*/*v*) phosphotungstic acid solution. After air drying at room temperature, the stained film was checked under TEM.

#### 2.2.6. Differential Scanning Calorimetry (DSC)

The thermal properties of SPNL were measured by DSC-1, utilizing a Mettler Toledo (8606 Greifensee, Switzerland) instrument, and under a nitrogen atmosphere. Each sample of about 10 mg was heated in an aluminum crucible from 25 to 200 °C at a scan rate of 10 °C/min. Each sample was run in triplicate. The results are presented as the average of the data obtained.

#### 2.2.7. PNL Solid Powders

The formulations and solid carriers (Neusilin US2, SYLOID 244 FP, Avicel PH 102, mannitolium and mannitol) were mixed thoroughly using a spatula with gentle heating being applied. In a typical experiment, a powder mixture of SPNL was prepared by mixing 100 mg of an SPNL with 650 mg of the solid carrier. At the same time, 150 µL of the LPNL was absorbed onto 750 mg of the solid carrier. The amounts of the CBD in the powder mixture were 5 ± 0.2% *w*/*w* and 2.5 ± 0.1% *w*/*w* for SPNL and the LPNL, respectively. The powder formulations were then stored in the dark at room temperature until further use. The samples were withdrawn at various intervals and analyzed for CBD content using HPLC.

### 2.3. Animal Study

#### 2.3.1. Aqueous PNL Dispersion Preparation for the Animal Study

The nano-dispersions of the SPNL and LPNL were prepared using 37 °C water and aimed to achieve CBD concentrations of 5 mg/mL. Usually, a fresh suspension is recommended within 2 h of the animal study. For the sake of comparison and to verify the efficiency of the SPNL, we used our previously reported PNL formulation as a reference standard [16]. This PNL will be referred to as CBD–PNL

#### 2.3.2. In Vivo Study with Rats

All experimental procedures were reviewed and approved by the Animal Experimentation Ethics Committee of the Hebrew University–Hadassah Medical School, Jerusalem (Ethical approval number: MD-22-17053-3). Male Wistar rats weighing 0.295–0.335 kg were used in all in vivo experiments and were obtained from Envigo.

A detailed study procedure is provided in Ref. [15]. In brief, before the surgery, animals were fasted for 12 h with free access to water. Following surgery, each animal was housed individually and allowed to recover overnight (12–18 h). During the recovery period, food was withheld while water remained available ad libitum. In the experimental phase, food was returned 4 h after oral administration of the tested formulation. Animals were randomly assigned to the different experimental groups.

Male Wistar rats (0.295–0.335 kg) were allocated to groups of either SPNL or LPNL formulations. Each member of the SPNL group was compared to one in the control group utilizing the liquid PNL formulation (CBD–PNL). The CBD–PNL group included 4 rats, while the LNPL group and the SPNL group included 7 rats, respectively. The CBD dose of each oral administration was constant at 15 mg/kg for all the groups. An aqueous dispersion was freshly prepared two hours before the experiment by vortex-mixing of the pre-concentrates in water pre-heated to 37 °C, forming homogeneous dispersions. The oral formulations were administered to the animals by oral gavage. Systemic blood samples (0.35 mL) were taken at 5 min pre-dose and at 0.33, 0.66, 1, 1.5, 2, 4, 6 and 8 h post-dose. To prevent dehydration, equal volumes of physiological solution were administered following each withdrawal of blood sample. Plasma was separated by centrifugation (4000 rpm, 10 min) and stored at −20 °C pending analysis.

Plasma aliquots of 150 µL were spiked with 10 µL of internal standard cannabigerol (CBG; 1 µg/mL). ACN (150 µL) was added to each test tube (tubes A) and vortex-mixed for 2 min. The extraction of CBD and CBG was performed by utilizing n-hexane (3 mL), which was added to each test tube (tube A), followed by 2 min. vortex-mixing. After centrifugation at 4000 rpm for 10 min, the n-hexane organic layer was transferred to fresh glass test tubes (tubes B) and evaporated to dryness (Vacuum Evaporation System, Labconco, Kansas City, MO, USA). Then, the B tubes were reconstituted in 80 µL of ACN:water (80:20). The resulting solution (80 µL) was injected into the HPLC-MS-MS system [comprising a Waters pump (600 controller), Waters autosampler (717 plus Auto-sampler), and Waters Micro-mass ZQ mass spectrometer (Waters Corporation, Milford, MA, USA)].

Data analysis (concentration vs. time data and pharmacokinetic parameters such T_max_ C_max_, and AUC) was performed using Microsoft Excel (Microsoft Office, Redmond, WA, USA) and plotted either in Excel or Origin software. All values are expressed as mean ± standard error of the mean (SEM), if not stated otherwise.

## 3. Results

We present the preparation and properties of the SPNL formulations. The blank SPNL appears as white solid wax which is non-greasy, and the maximum amount of CBD dissolved in SPNL is 40% *w*/*w*. Upon addition of the CBD, the physical texture of the SPNL remains like the blank; however, the formulation color changes to off-white. The CBD content using HPLC in the final SPNL formulation is 40 ± 2% *w*/*w*. On the other hand, the blank LPNL formulations appear as transparent liquid mixtures. Upon addition of the 25% *w*/*v* of the CBD, the transparency in the formulation is still maintained, whereas the color turns to light yellow due to the dissolved drug. The CBD content when using HPLC in the final LPNL formulation was 25 ± 0.2% *w*/*v*.

**Figure 1:**
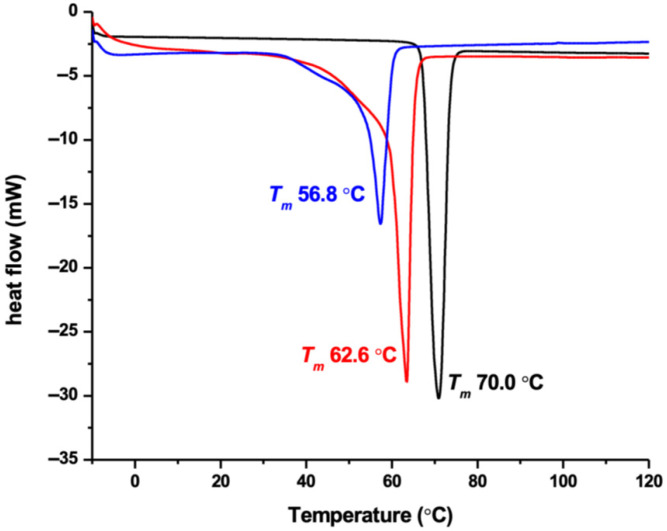
DSC analysis of the CBD alone (black line), blank SPNL (red line), and 40% *w*/*w* CBD-loaded SPNL (blue line).

DSC analysis of the SPNL showed a single melting temperature (T_m_) for both the blank and CBD-loaded SPNL. As shown in Figure 1, T_m_ is 70 °C, whereas the blank and CBD-loaded SPNL show T_m_ at 62.6 and 56.8 °C, respectively.

### 3.1. Dispersion Analysis

**Figure 2:**
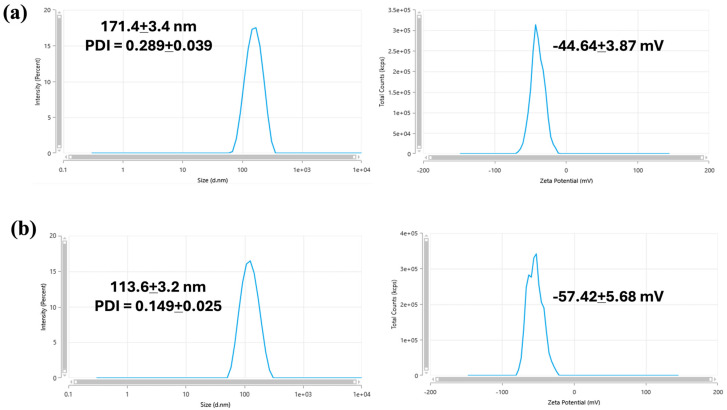
DLS and surface charge (zeta potential) of aqueous dispersions of SPNL and LPNL in DDW at 37 °C. The numbers represent an average of the triplicate analysis for each sample.

In sum, 200 mg of SPNL or LPNL formulations were dispersed into 5 mL of pre-heated 37 °C DDW. Then, 20 µL of the concentrated dispersion was further diluted into 1 mL of the DDW and examined using DLS at 37 °C. As shown in Figure 2, the sizes of the aqueous dispersions of the SPNL and LPNL were 171.4 ± 3.4 nm (PDI: 0.289 ± 0.039) and 113.6 ± 3.2 nm (0.149 ± 0.025), respectively. The zeta potentials of the dispersions were −44.64 ± 3.87 mV and −57.42 ± 5.68 mV for the aqueous dispersions of the SPNL and LPNL, respectively. It should be noted that the size of the nano-dispersion remained similar (<200 nm) for both the PNLs in PBS, as well, at 37 °C. Apparently, nano-dispersions of 147.5 ± 6.3 nm (PDI: 0.211 ± 0.059) and 114.1 + 2.5 nm (PDI: 0.149 ± 0.025) were obtained for SPNL and LPNL in PBS, respectively.

The stability of the aqueous dispersion of the PNLs in PBS was confirmed by keeping the concentrated dispersions at room temperature and 37 °C for 24 h. When stored at room temperature, the sizes of the nano-dispersions after 24 h were 227.0 ± 38.2 nm (PDI: 0.399 ± 0.038) and 119.9 ± 2.1 nm (PDI: 0.125 ± 0.029) for the SPNL and LPNL. On the other hand, as shown in Table 2, the sizes of the nano-dispersions after 24 h were <150 nm in both the SPNL and LPNL dispersions stored at 37 °C for 24 h. The homogeneity in the nano-dispersions in PBS stored at room temperature and at 37 °C for 24 h was determined. For this, 200 mg of each formulation was dispersed in 5 mL PBS, so uniform SPNL and LPNL aqueous dispersions would show 16 mg/mL and 10 mg/mL CBD. We see a decrease in the CBD amounts for the dispersion stored at room temperature for 24 h. This is due to sedimentation of the solid nanoparticles. These obtained 18.6 ± 1.8 mg/mL and 8.0 ± 0.4 mg/mL CBD in aqueous dispersions of SPNL and LPNL stored at room temperature for 24 h. On the other hand, the sample dispersions stored at 37 °C for 24 h show less deviation in the CBD content. The CBD amounts matched with the calculated amounts for homogeneous aqueous dispersions of SPNL and LPNL at 37 °C.

### 3.2. TEM Analysis of the PNL Aqueous Dispersion

**Figure 3:**
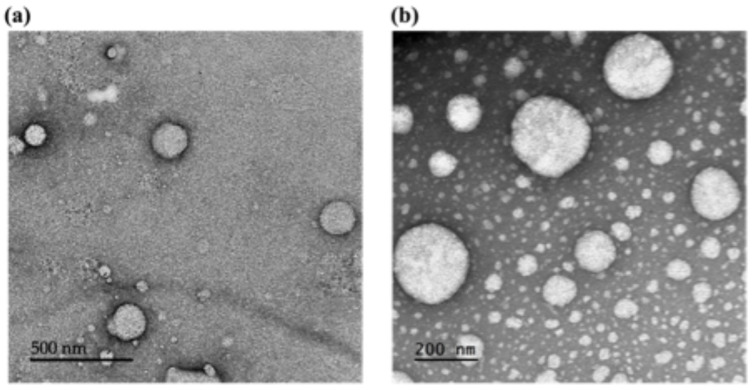
TEM images of (**a**) SPNL and (**b**) LPNL aqueous dispersions. Analysis was performed at room temperature.

TEM analysis of the SPNL and LPNL aqueous dispersions shows the formation of spherical particles with solid cores with sizes < 200 nm (Figure 3). Uniformity in the sample is seen, as all the particles are spherical in shape. No signs of aggregation or drug crystals are seen. The TEM results are in accordance with the DLS findings, and the size of the nano-dispersion is also <200 nm.

### 3.3. Storage Stability of SPNL and LPNL

The stability of the CBD-loaded PNL formulations stored in closed vials for more than 3 months at room temperature in the dark was examined. The loss in the CBD content in both SPNL and LPNL after 3 months of storage was ≤2% *w*/*w*. After 3 months at room temperature, 38.1 ± 0.8% *w*/*w* and 23.3 ± 0.7% *w*/*v* CBD were obtained for the SPNL and LPNL.

### 3.4. Storage Stability of PNL Formulation with Solid Carrier

In reports in the literature, a solid SNEDDS powder is achieved by absorbing the SNEDDS onto popular solid carriers such as mannitol, microcrystalline cellulose, and mesoporous silica particles such as Syloid 244 FP and Neusilin US2 [19]. Solid-SNEDDS exceeds the storage properties of the lipid-based formulations and is suitable for successful processing into capsules or tablets with adequate flow and compressibility [23]. The long-term storage stability of the formulations was checked with the solid carrier at room temperature. SPNL and LPNL showed losses in the neighborhood of ~60% CBD content with silica carriers, such as Neusilin US2 and SYLOID 244 FP, after 3 months. On the other hand, with the Avicel PH 102, mannitolium and mannitol, the stability of the SPNL and LPNL mixtures is incredible with no loss in CBD noted.

### 3.5. Pharmacokinetic (PK) Assessment of PNLs in Rats

**Figure 4:**
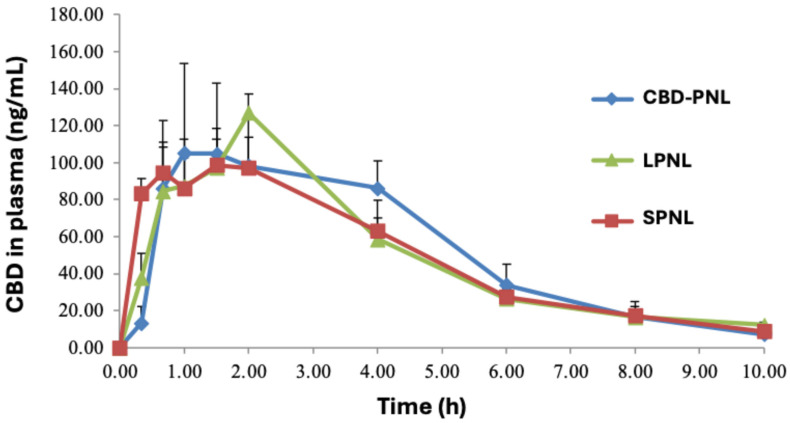
PK profile of plasma levels of CBD vs. time plot following PO administration of 15 mg/kg given in the form of CBD–PNL (blue ♦), LPNL (green ▲) and SPNL (red ■), each in aqueous dispersion.

The CBD amounts in the rat plasma as a factor of the time profile for the SPNL were obtained after oral administration to rats at a dose of 15 mg/kg (Figure 4). The corresponding AUC, C_max_, T_max_ and linear terminal slope (K) parameters for SPNL are listed in Table 3. As seen in Figure 4, the SPNL formulations were well-absorbed and resulted in significant CBD concentrations in the plasma. The CBD concentration in the blood shows a C_max_ of 122 ± 26 ng/mL. On the other hand, a C_max_ of 140 ± 31 ng/mL was seen for the LPNL formulation. For the CBD–PNL, a C_max_ of 146 ± 62 ng/mL was perceived. Based on the pharmacokinetics results in rats, CBD has a rapid elimination rate with significant reduction of blood plasma levels occurring within a 10-h timeframe. The C_max_ values indicate that both SPNL and LPNL are associated with good oral bioavailability of the CBD; this is consistent with our previous investigations with PNLs, which show an increase in the drug C_max_ to 137 ± 43 (ng/mL) [16].

### 3.6. Improvisation Utilizing SPNL for Other Lipophilic Drugs

As stated above, SPNL was designed exclusively for CBD. SPNL did not show a uniform nano-dispersion when a drug other than CBD was used. Therefore, the SPNL formulation was optimized, in order that the technology itself would not be inherently restricted to CBD (Table 4i). The addition of polyethylene glycols (PEGs) to the SPNL composition not only supported drug dissolution but also quickened the dispersion time of the SPNL. As shown in Table 4ii, the addition of various PEGs to the SPNL composition helps to maintain the dispersion size of the formulation at <200 nm.

## 4. Discussion

The present work reports an improved version of previously described PNL formulations. Unlike the regular PNLs or SNEDDS reported in the literature, the SPNL are solid waxes at room temperature, with a melting temperature of >50 °C. SPNL contain solid lipids that have melting points that are between ~60 and 65 °C, which is very close to the melting point of CBD (~70 °C). This similarity in melting points will allow CBD to exist in the amorphous state within the SPNL. The amorphous CBD is preferable and will eventually be uniformly distributed in the SPNL formulation. Similar phenomena are seen with CBD-loaded solid lipid nanoparticles, in which the characteristic peaks for crystalline CBD form were not observed in the lipid nanoparticles, suggesting that the drug was effectively dissolved in an amorphous state within the lipid matrix [25,26,27]. We also show that the compatibility of the developed SPNL with other lipophilic drugs is strong and can be achieved by the addition of PEGs or similar co-solvents within the developed composition. On the other hand, we also prepared stable liquid PNL formulations, CBD-loaded LPNL with 25% *w*/*v* drug content. The stability of the CBD in the lipid systems (e.g., SNEDDS) is still unclear in the available literature [28,29]. However, in our study, the CBD remained soluble in both the SPNL and LPNL formulations after preparation at room temperature. No signs of the aggregation/crystallization were seen later upon long-term storage.

Achieving a self-emulsification system with high CBD-loading that forms a stable nano-dispersion (≤200 nm) is always challenging. There are a limited number of works in the available literature that describe self-emulsifying formulations with varying amounts of CBD. SNEDDS formulations have been loaded with 20–21% CBD and achieved a 200 nm dispersion size [11,12]. Balenzano et al. [13] created a SNEDDS formulation with 10% *w*/*v* CBD and a dispersion size < 100 nm. Kok et al. [10] prepared 10% CBD SNEDDS with a dispersion size of ~50 nm. Wu et al. [8] reported SNEDDS with 12% *w*/*w* CBD and a dispersion size of ~70 nm. Grifoni et al. [9] reported SNEDDS containing 2% *w*/*w* CBD with dispersion size of >30 nm. Cherniakov et al. [16] prepared a PNL formulation with 3% CBD with a dispersion size of <50 nm. In comparison, our SPNL formulation can solubilize up to 40% *w*/*w* of the CBD and produce a solid nanoparticle of <200 nm. This high solubility of the CBD is unmatched and has not been reported in any of SNEDDS systems to date. Similarly, the LPNL is also associated with a high CBD loading and shows a dispersion size of <200 nm.

The shelf-life of the SPNL and LPNL at room temperature was tested for three months. Both of the formulations showed good stability when stored at room temperature for 3 months. The aqueous dispersion was prepared from PNL samples that were stored at room temperature for 3 months. It showed a nano-dispersion of <200 nm. This is an indication of the stability of our PNL formulations. As reported in the literature, it is desirable that a stable CBD–SNEDD formulation should show a dispersion similar to the dispersion size of the freshly prepared formulation [8]. We did not observe any significant decrease in the CBD content in either of the SPNL or LPNL formulations kept within room temperature storage conditions for more than 3 months.

Solid-SNEDDS systems are often preferred over the SNEDDS formulation because of the ease of preparation, storage, and transportation. The solid-SNEDDS are free-flowing powders with a non-oily texture and are readily usable in solid dosage forms such as being filled into soft gelatin capsules or compressed into oral tablet form [21,30]. The solid carriers used in the solid-SNEDDS preparation are inert and supposedly do not pose any stability issues relative to the drug in its powdered form [23,31]. In the literature, there is little discussion of the stability of the drugs in solid-SNEDDS. Usta et al. [32] prepared BOS-loaded solid-SNEDDS using Neusilin^®^ US2 and investigated formulation stability at 25 ± 2 °C/60 ± 5% RH conditions. The authors show that the drug remains stable at room temperature for 6 months. SPNL and LPNL are composed of several kinds of solid lipids, solvents, and surfactants; hence, it is likely that there might exist interactions between the excipients and solid carriers. Upon examination, the lack of PNL stability with Neusilin US2 and SYLOID 244 FP was noted. On the other hand, no loss in the CBD content was observed with solid carriers that do not contain silicates in any form, for example, carriers such as Avicel PH 102 or mannitol.

SNEDDS provides the advantage of faster CBD absorption upon oral delivery. This determination implies that the SNEDDS facilitates quicker drug dissolution and absorption across the intestinal membrane, which is represented through the T_max_ values [33]. There is a need for a fast-absorbing formulation, because oral delivery of the CBD in oils generally shows a delayed absorption, with T_max_ ~4 h or more [8,34]. A shorter T_max_ for CBD represents a faster drug absorption and better SNEDD formulation. Previously reported PNL formulations have shown values for T_max_ of 1–1.5 h for the CBD after oral administration in rats [15,16,35]. In support of this, Sandmeier et al. [11] also obtained the T_max_ of ~2 h for its CBD–SNEDDS formulations in rats. Kok et al. [10] obtained a T_max_ of ~1 h for its CBD–SNEDDS formulations in rats. Wu et al. [8] also observed a shorter T_max_, ~1 h, for their CBD–SNEDDS formulation. As shown in Figure 4, the PK evaluations of the SPNL and LPNL show T_max_ values of ~1 and 2 h, respectively. This indicates that the SPNL and LPNL formulations are absorbed faster upon administration. The C_max_ values of SPNL and LPNL were calculated to be 122 ± 26 and 140 ± 31 ng/mL, which are similar to that for CBD–PNL (C_max_, 146 ± 62 ng/mL) and comparable to our previous results with the other PNL systems [15,16].

Overall, the developed SPNL formulation has an oral bioavailability of the CBD which is similar to that of the CBD–PNL standard formulation. We have created an improved PNL formulation with enhanced physical stability without compromising the rapid onset of absorption of the CBD. Our developed SPNL formulation differs in several ways from the previously reported liquid PNLs or SNEDDS systems, as it does not contain any organic solvent. All the CBD–SNEDDS and PNL formulations reported to date require co-solvents such as, benzyl alcohol, PEG-23-laurylyether, ethyl lactate, propylene glycol, ethanol, n-methyl-2-pyrrolidone, or transcutol in order to prevent drug crystallization or precipitation [8,9,10,11,12,13,15,16,17]. Avoiding the use of organic solvents or keeping their amounts low will reduce the risk of toxicity to a great extent. Overall, we believe that SPNL can potentially reduce adverse solvent effects. This means improved safety outcomes, particularly in vulnerable populations [36,37].

## 5. Conclusions

This study presents the development of solid PNL (SPNL) for the first time. The practical distinction between the SPNL and SNEDDS systems is a fundamental difference in the use of lipidic excipients, with the SPNL solid having a melting point ≥ 50 °C. In parallel, a modified liquid PNL system, LPNL, was also developed; it had a better solubilizing capability for the CBD. A maximum solubility of the CBD in both the SPNL and LPNL was achieved amounting to 40% *w*/*w* (and 25% *w*/*v* is extraordinary), along with the desired uniform nano-dispersion of <200 nm. Both formulations are stable, with no signs of drug precipitation, drug loss, or phase separation seen for 3 months at room temperature. The bioavailability of CBD following the administration of the SPNL and LPNL formulations in rats showed results comparable with the previously reported PNL formulation. The SPNL and LPNL formulations can be readily transformed into solid powder; however, there are concerns about the use of mesoporous silica particles. Their use should be avoided.

In conclusion, we have presented a novel SPNL as an unconventional formulation for CBD, one exceptionally different from those reported in the literature. This SPNL preparation is easy and can even be manufactured using simple and economical equipment, making large-scale production feasible. Since SPNL is composed of GRAS-status components, its approval for the conducting of clinical studies or as a commercial product can be easily achieved.

## Figures and Tables

**Table 1:** Composition of blank PNLs.

Material	% *w*/*w*
**Blank-SPNL**	
IMWITOR^®^ 900 K	40
Tween 80	10
Kollipor RH 40	10
Geleol™ Mono and Diglycerides NF	20
Labrafil^®^ M 1944 CS	20
**Blank-LPNL**	
Sesame oil	12
Kollipor RH 40	20
Span 80	20
Tween 20	20
Solutol HS 15	8
Propylene glycol	20

Details of each compound are given above in Section 2.1, Materials.

**Table 2:** DLS comparison of the size for the freshly prepared SPNL or LPNL dispersion in PBS at 37 °C and dispersion after 24 h at room temperature.

Sample	Freshly Prepared	24 h, RT	24 h, 37 °C
SPNL	171.4 ± 3.4 nm (PDI: 0.289 ± 0.039)	227.0 ± 38.2 nm (PDI: 0.399 ± 0.038)	124.9 ± 0.4 nm (PDI: 0.169 ± 0.043)
LPNL	113.6 ± 3.2 nm (PDI: 0.149 ± 0.025)	119.9 ± 2.1 nm (PDI: 0.125 ± 0.029)	128 ± 3.0 nm (PDI: 0.206 ± 0.023)

**Table 3:** PK parameters of plasma levels of CBD vs. time plot following PO administration of 15 mg/kg given in the form of CBD–PNL, LPNL and SPNL nano-dispersion, each in water.

Mean ± SD	CBD–PNL	LPNL	SPNL
AUC_0–10_ (ng*h/mL)	525 ± 153	485 ± 162	501 ± 85
AUC_0–∞_ ^#^ (ng*h/mL)	545 ± 147	527 ± 172	561 ± 76
C_max_ (ng/mL)	146 ± 62	140 ± 31	122 ± 26
K (terminal slope 1/h)	0.43 ± 0.02	0.33 ± 0.13	0.35 ± 0.12
T_0.5_ (h)	1.61 ± 0.01	2.36 ± 0.79	2.19 ± 0.87
F_abs_^$^ (%) (Absolute bioavailability)	12.1 ± 3.3	11.70 ± 3.82	12.45 ± 1.69
T max (median, range)	3 (1, 4)	2 (0.67, 2)	1 (0.67, 2)

^#^ AUC_0–∞_ is calculated for infinity by using the terminal slope. ^$^ F_abs_ = % of absolute bioavailability was calculated using data for CBD in PNL by IV administration.

**Table 4:** (**i**) Compositions of blank-SPNL with various PEGs. (**ii**) Effects of PEGs on the sizes of the aqueous dispersions of 10% *w*/*w* CBD-loaded SPNL.

(i)		(ii)			
Materials	PEG Type	PEG Type	% *w*/*w* PEG	Size, nm	PDI
IMWITOR^®^ 900 K	PEG-1k	PEG-1k	10	110.2 ± 0.5	0.24 ± 0.02
Tween 80	PEG-4.5k	PEG-4.5k	10	139.3 ± 1.5	0.23 ± 0.05
Kollipor RH 40	PEG-4.5k	PEG-4.5k	20	124.5 ± 3.2	0.20 ± 0.02
PEG-1k/-4.5k/-6k	PEG-6k	PEG-6k	10	146.9 ± 4.8	0.27 ± 0.06
Labrafil^®^ M 1944 CS	30				

The SPNL composition with PEG-1k was further tested with various drugs because of its fast-emulsifying properties. In this case, 10% *w*/*w* of the lipophilic drugs (curcumin, ibuprofen, 7-Nitroindazole (7-NI) and oxybenzone) in SPNL were prepared and tested. DSC analysis shows that the melting points are between 58 and 60 °C for all formulations of drugs loaded with SPNL. All of the formulations showed the desired dispersion sizes of ≤200 nm (curcumin: 243.1 ± 41.6 nm, PDI: 0.58 ± 0.16; ibuprofen: 154.1 ± 6.1 nm, PDI: 0.33 ± 0.07; 7-NI: 154.3 ± 2.6 nm, PDI: 0.24 ± 0.01; and oxybenzone: 142.1 ± 1.4 nm, PDI: 0.24 ± 0.01, in water, respectively.

## Data Availability

The raw data supporting the conclusions of this article will be made available by the authors on request.
